# Characterization, kinetic, and isotherm data for Cr(VI) removal from aqueous solution by Cr(VI)-imprinted poly(4-VP-co-MMA) supported on activated Indonesia (Ende-Flores) natural zeolite structure

**DOI:** 10.1016/j.dib.2018.01.076

**Published:** 2018-02-02

**Authors:** Yantus A.B. Neolaka, Ganden Supriyanto, Handoko Darmokoesoemo, Heri Septya Kusuma

**Affiliations:** a Chemical Education Department, Faculty of Education and Teachers Training, University of Nusa Cendana, Kupang, 85001, Nusa Tenggara Timur, Indonesia; b Department of Chemistry, Faculty of Science and Technology, Airlangga University, Mulyorejo, Surabaya 60115, Indonesia; c Department of Chemical Engineering, Faculty of Industrial Technology, Institut Teknologi Sepuluh Nopember, Surabaya 60111, Indonesia

**Keywords:** Activated natural zeolite, Poly(4-VP-co-MMA), Cr(VI) adsorption

## Abstract

The adsorption performance of Cr(VI) on the Cr(VI)-imprinted poly(4-VP-co-MMA) (IIP) supported on Activated Indonesia (Ende-Flores) natural zeolite (ANZ) structure for Cr(VI) removal from aqueous solution have been studied. Cr(VI)-imprinted-poly(4-VP-co-MMA)-ANZ (IIP-ANZ) was synthesized using Cr(VI) as a template, 4-vinylphiridine (4-VP) as a complex agent, methyl methacrylate (MMA) as a monomer agent, ethylene glycol dimethylacrylate (EGDMA) as cross-linker and benzoyl peroxide (BPO) as an initiator. XRD, FTIR, SEM-EDX and BET was performed to characterize the synthesized materials. The maximum adsorption capacity was 2.431 mg/g adsorbent at pH 2, contact time of 30 min, under 303 K respectively. Five kinetic and four isotherm models were used to find out the reaction rate of Cr(VI) adsorption processes on this adsorbent. Under the competitive condition, the adsorption capacity of this adsorbent for Cr(VI) is greater than Cr(III), Mn(II) or Ni(II) ions but it less selective if present of Pb(II) ion. Moreover, the reusability of the IIP-ANZ was tested for five times and no significant loss in adsorption capacity observed.

**Specifications Table**

**Table:** 

Subject area	*Chemical Engineering*
More specific subject area	*Adsorption*
Type of data	*Table, image, figure*
How data was acquired	– *The uptake of Cr(VI) by the adsorbent (q*_*e*_*) was determined based on the subtraction of the initial and final concentration of adsorbate* – *Fourier transform infrared (FTIR) spectroscopy (Shimadzu, FTIR 8000 Series), scanning electron microscopy with energy dispersive X-ray (SEM-EDX) spectroscopy (JEOL, JMS 5600, Tokyo, Japan), X-ray diffraction (Shimadzu, XRD-6000), Quantachrome Instruments NOVA 1200 (High-Speed Gas Sorption Analyzer Versions 10.0 – 10.03) was used for determine the characteristics of the adsorbent* – *The Cr(VI) concentration measurement was performed by UV–vis spectroscopy (Shimadzu, UV-1240)*
Data format	*Analyzed*
Experimental factors	– *To synthesize Cr(VI)-imprinted-poly(4-VP)-ANZ (IIP-ANZ), Cr(VI) was used as a template, 4-vinyl pyridine (4-VP) was used as a complex agent, methyl methacrylate (MMA) as a monomer agent, ethylene glycol dimethacarylate (EGDMA) as crosslinker and benzoyl peroxide (BPO) as an initiator* – *For comparison, NIP-4-VP-co-MMA-ANZ (non ion imprinted polymer) (NIP-ANZ) was also prepared using an identical procedure without the addition of dichromate ion* – *Data of IIP-ANZ were acquired for Cr(VI) removal from aqueous solution*
Experimental features	*IIP-ANZ for Cr(VI) adsorption from aqueous solution*
Data source location	*Airlangga University, Surabaya, Indonesia*
Data accessibility	*Data are accessible with the article*

**Value of the data**

• The newly synthesized adsorbent has a good potential application in related of wastewater treatment or to use in solid phase extraction
• The isotherm, kinetic, and thermodynamic data will be informative and useful for predicting and modeling the adsorption capacity and mechanism of chromium removal by the adsorbent
• The acquired data will be advantageous for the scientific community wanting to scale up and design an adsorption column with IIP-ANZ as medium for the removal of Cr(VI)-containing waters or wastewaters

## 1 Data

**Fig. 1:**
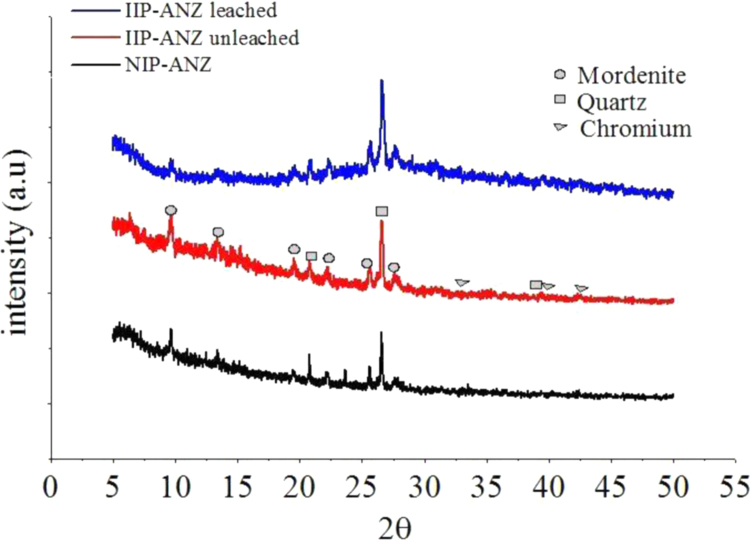
XRD patterns of IIP-ANZ unleached, IIP-ANZ leached and NIP-ANZ.

**Fig. 2:**
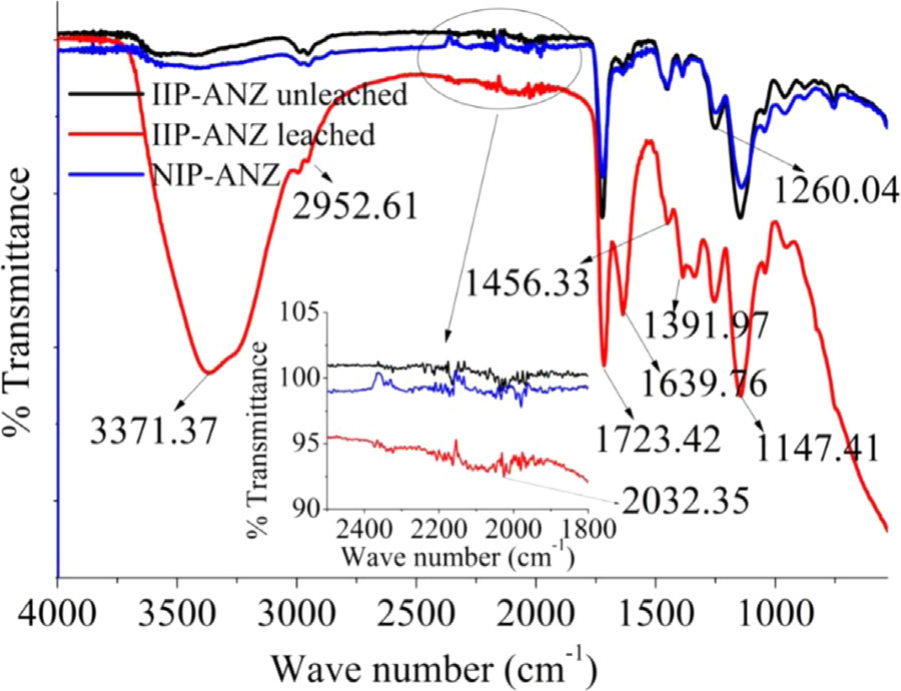
The FTIR spectra of IIP-ANZ unleached, IIP-ANZ leached and NIP-ANZ.

**Fig. 3:**
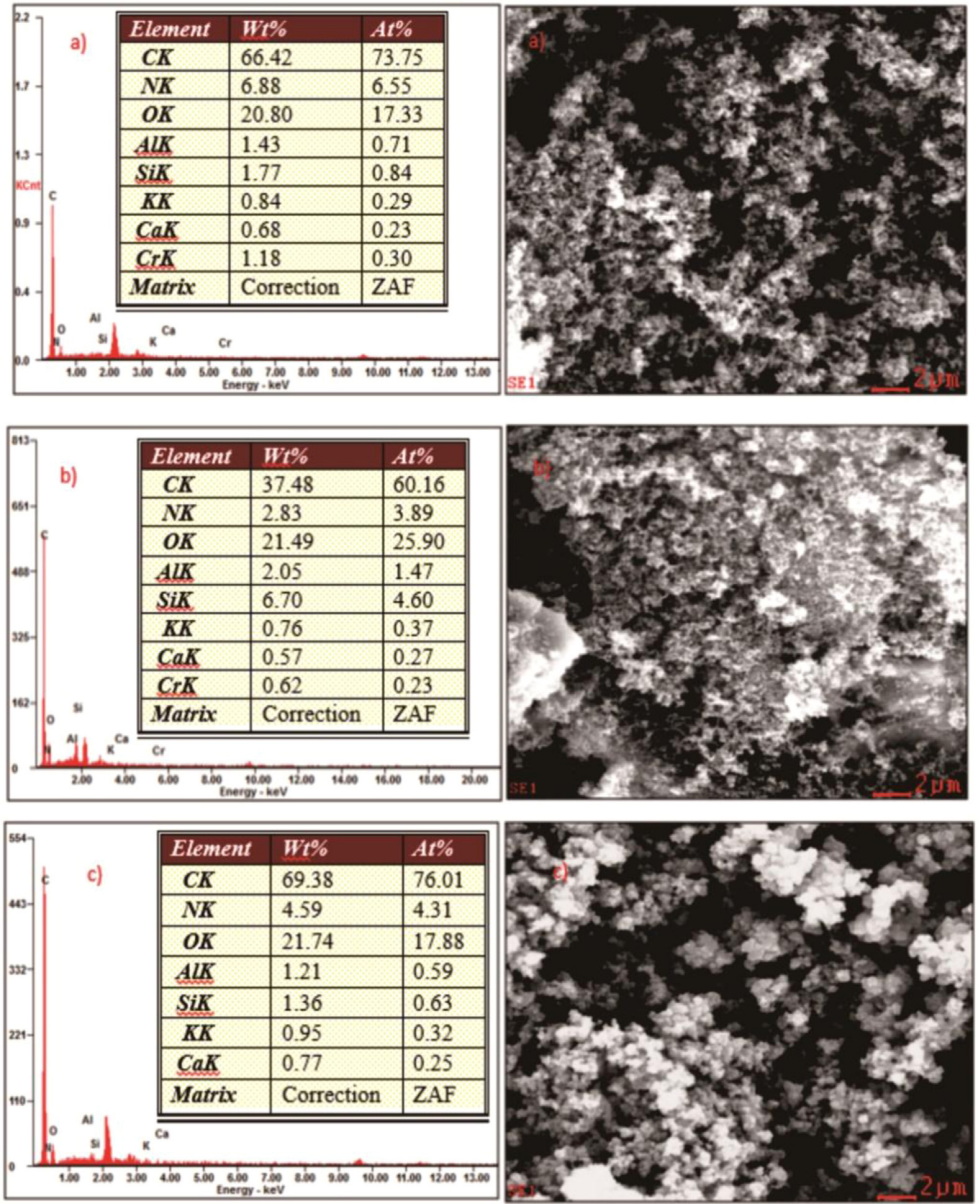
SEM-EDX analysis of: (a) IIP-ANZ unleached, (b) IIP-ANZ leached and (c) NIP-ANZ.

**Fig. 4:**
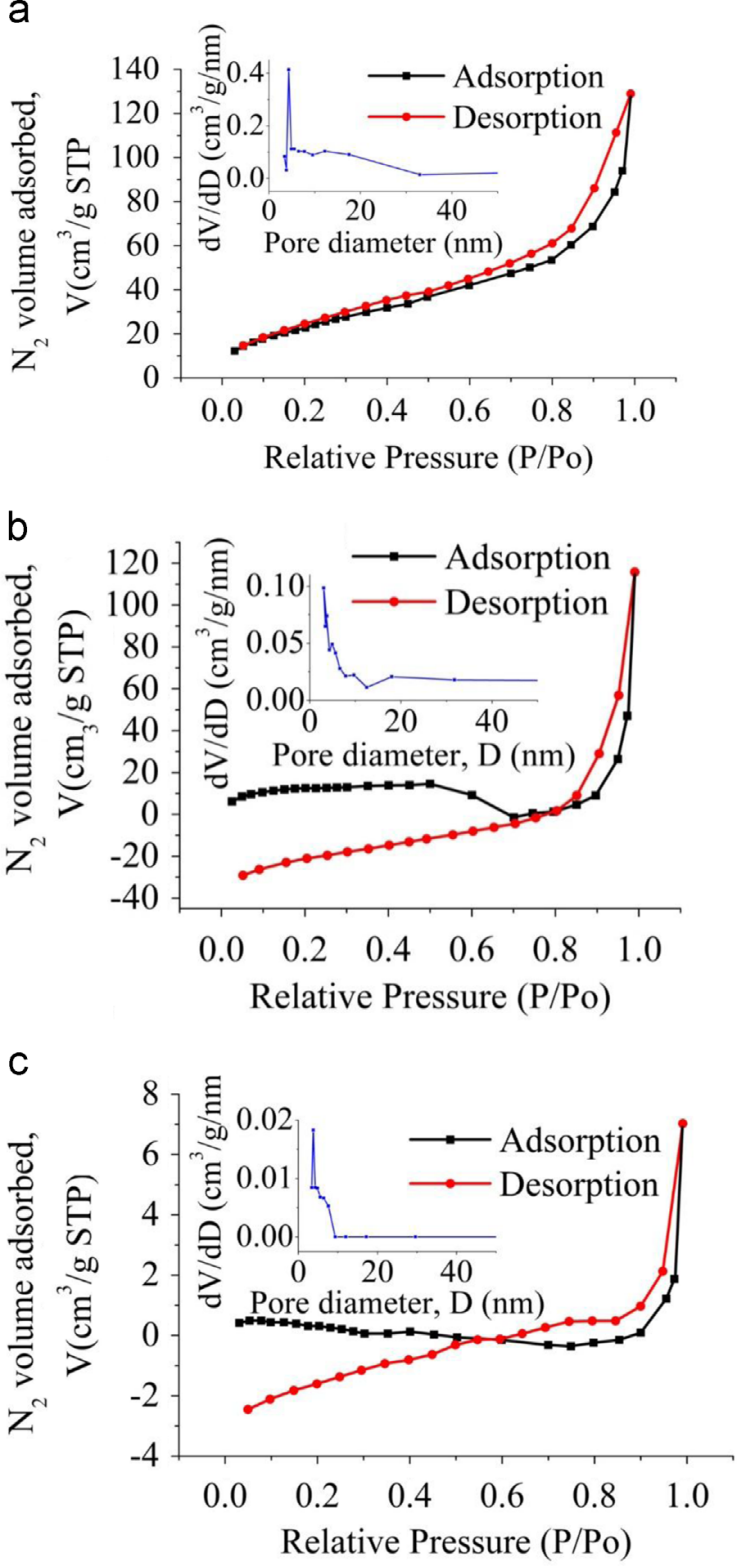
BET and BJH analysis of: (a) IIP-ANZ unleached, (b) IIP-ANZ leached and (c) NIP-ANZ.

**Fig. 5:**
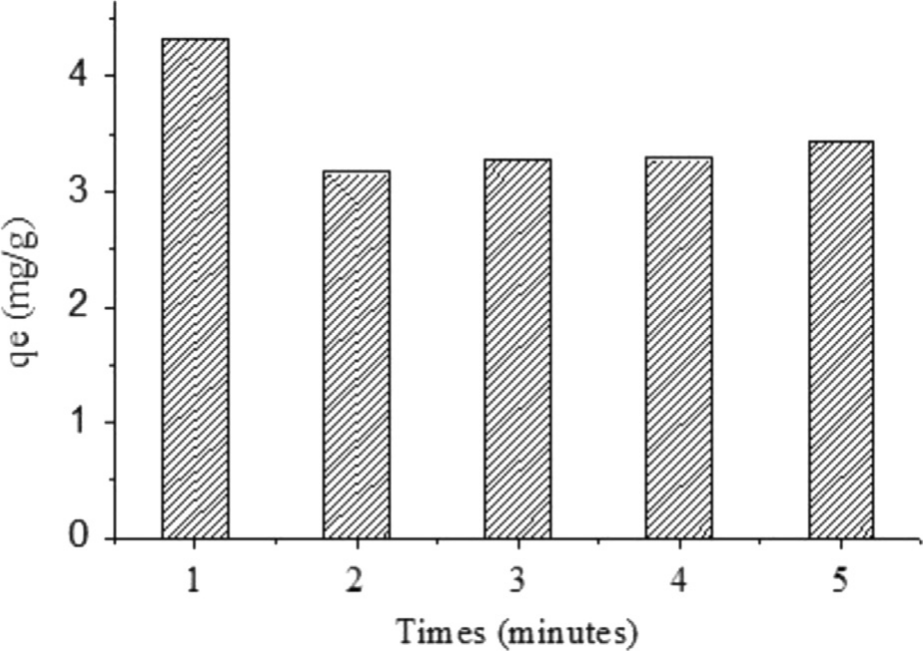
Reusability of IIP-ANZ.

The XRD patterns of IIP-ANZ unleached, IIP-ANZ leached and NIP-ANZ are shown in Fig. 1. The FTIR of IIP-ANZ unleached, IIP-ANZ leached and NIP-ANZ at wave numbers from 400 to 4000 cm^−1^ are given in Fig. 2. The results of the SEM-EDX analysis for IIP-ANZ unleached, IIP-ANZ leached and NIP-ANZ are shown in Fig. 3. Characterization of BET and BJH for IIP-ANZ unleached, IIP-ANZ leached and NIP ANZ was presented in Fig. 4 and Table 1. The pH of zero point charge, pH_ZPC_, for IIP-ANZ leached and NIP-ANZ obtained is shown in Table 1. The optimum condition for Cr(VI) adsorption by IIP-ANZ are presented in Table 2. The kinetics and isotherms parameters for the adsorption of Cr(VI) by IIP-ANZ and NIP-ANZ were estimated using models listed in Table 3, Table 4. The kinetics, isotherm, and thermodynamic parameters for the adsorption of Cr(VI) by IIP-ANZ and NIP-ANZ is presented in Table 5, Table 6, Table 7. Adsorption capacities of IIP-ANZ and NIP-ANZ in the presence of competitive ions such as Cr(VI)/Pb(II), Cr(VI)/Mn(II), Cr(VI)/Ni(II) and Cr(VI)/Cr(III) was studied in a batch system and the result was presented in Table 8 and the calculated K_d_, k and k’ parameters are given in Table 9. the reusability of the IIP-ANZ, the adsorption-desorption cycle was repeated five times, and the results are shown in Fig. 5.

**Table 1:** Physical parameters of IIP-ANZ unleached, IIP-ANZ leached and NIP-ANZ.

Samples	BET surface area^a^ (m^2^/g)	Total pore volume^b^ (cm^3^/g)	Micropore volume^c^ (cm^3^/g)	Mesopore volume (cm^3^/g)	Average pore Diameter (nm)	pH_ZPC_
IIP-ANZ unleached	32.752	0.199	0.142	0.057	4.158	–
IIP-ANZ leached	39.065	0.242	0.179	0.063	3.496	2.23
NIP-ANZ	0.180	0.038	0.010	0.028	3.880	3.19

a Multi point BET.

b Total volume pore total at *P*/*P*_0_ = 0.99005 (IIP-ANZ unleached), 0.99048 (IIP-ANZ leached) and 0.99098 (NIP-ANZ).

c Mesopore volume = Total pore volume – Micropore volume.

**Table 2:** The optimum condition for Cr(VI) adsorption by IIP-ANZ (The concentration of Cr(VI) solution is 14 mg/L).

Parameters	Variation range	Optimum value	*q*_e_ (mg/g)	Adsorption efficiency (%)
Adsorbent amount (g)	0.01–0.20	0.1	2.431	97.23
pH	1–9	2		
Time (min)	0–120	30		
Temperature (K)	303–343	303

**Table 3:** Kinetic models/equations used in this data article.

Kinetic models	Equation	References
Pseudo first order	$\log\left(q_{e}-q_{t}\right)=\ln q_{e}-k_{1}t$	[1]
Pseudo second order	$\frac{t}{q_{t}}=\frac{1}{k_{2}q_{e}^{2}}+\frac{1}{q_{e}}t$	[2]
Elovich	$q_{t}=\left(\frac{1}{\beta }\right)\ln\left(\alpha \beta \right)+\left(\frac{1}{\beta }\right)\ln t$	[2]
Intraparticle diffusion	$q_{t}=k_{i}t^{0.5}+C$	[3]
Bangham	$\log\log\left(\frac{C_{o}}{C_{o}-q_{t}m}\right)=\log\left(\frac{\mathrm{km}}{2.303V}\right)+\alpha \log t$	[4]

**Table 4:** Isotherm models/equations used in this data article.

Isotherm models	Equation	References
Langmuir	$\frac{C_{e}}{q}=\frac{1}{Kq_{\max}}+\frac{1}{q_{\max}}C_{e}$	[5]
Freundlich	$\log q=\log K_{F}+\frac{1}{n}\log C_{e}$	[5]
Tempkin	$q_{e}=\frac{\mathrm{RT}}{B_{T}}\ln A_{T}+\frac{\mathrm{RT}}{B_{T}}\ln C_{e}$	[6]
Dubinin–Kaganer–Radushkevich (DKR)	$\ln q_{e}=\ln q_{s}-k_{\mathrm{ad}}\varepsilon^{2}$	[6]

**Table 5:** The kinetics data for Cr(VI) adsorbed onto IIP-ANZ and NIP-ANZ.

Kinetic models	Parameters	Adsorbent	
		IIP-ANZ	NIP-ANZ
Pseudo-first order	*k*_1_/10^−3^ min^−1^	0.0382	0.239
	*q*_e_. mg g^−1^	4.089	6.144
	*R*^2^	0.967	0.662
Pseudo-second order	*k*_2_/10^−3^ g mg^−1^ min^−1^	1.100	1.382
	*q*_e_. mg g^−1^	0.932	1.066
	*h*/10^-2^ mg g^−1^ min^−1^	0.957	1.573
	*R*^2^	0.993	0.995
Intraparticle Distribution	*k*_i_/10^−2^ mg g^−1^ min^-0.5^	0.667	0.193
	*C*. mg g^-1^	2.054	1.815
	*R*^2^	0.975	0.911
Bangham	km (mL/(g/L))	4.895	3.414
	*α*	2.214	1.409
	*R*^2^	0.981	0.872
Elovich	*α* /10^−2^ mg g^−1^ min^−1^	2.787	372.680
	*β*. g mg^-1^	0.825	2.910
	R^2^	0.974	0.872

**Table 6:** Isotherm parameters for adsorption Cr(VI) on IIP-ANZ and NIP-ANZ.

Isotherm adsorption models	Parameters	Adsorbent	
		IIP-ANZ	NIP-ANZ
Langmuir	*Q*_max_ (mg/g)	-0.112	0.956
	*K*_L_ (L/mg)	0.251	-1.320
	*R*^2^	0.637	0.953
Freundlich	*n*	-1.910	3.924
	*K*_F_ (mg kg^−1^)	2.647	2.731
	*R*^2^	0.592	0.655
Tempkin	*K*_t_	0.027	18.547
	*b* (kJ/mol)	0.260	0.940
	*R*^2^	0.855	0.659
Dubinin–Kaganer–Radushkevich (DKR)	*X*_m_ (mg/g)	1.000	4.432
	*β* (kJ/mol)	8 × 10^–11^	−2 × 10^−10^
	*R*^2^	0.386	0.718

**Table 7:** Results of thermodynamic experiment for adsorption Cr(VI) onto IIP-ANZ and NIP-ANZ (The concentration of Cr(VI) solution is 14 mg/L).

T (K)	∆*G*° (kJ/mol)		∆*H*^o^ (kJ/mol)	
	IIP-ANZ	NIP-ANZ	IIP-ANZ	NIP-ANZ
303	-6.153	-55.483	-3.606	6.110
313	-6.237	-57.516	∆*S*^o^ (kJ/mol)	
323	-6.321	-59.549		
333	-6.405	-61.581	IIP-ANZ	NIP-ANZ
343	-6.490	-63.614	0.008	0.203

**Table 8:** Competitive adsorption of Cr(VI)/Pb(II), Cr(VI)/Mn(II), Cr(VI)/Ni(II) and Cr(VI)/Cr(III) on the IIP-ANZ and NIP-ANZ.

Ion		*q*_e_ (mg/g)	
		IIP-ANZ	NIP-ANZ
Cr(VI)/Pb(II)	Cr(VI)	2.510	1.831
	Pb(II)	0.464	0.606
Cr(VI)/Mn(II)	Cr(VI)	2.831	7.099
	Mn(II)	-0.163	-2.402
Cr(VI)/Ni(II)	Cr(VI)	7.738	6.996
	Ni(II)	3.984	1.826
Cr(VI)/Cr(III)	Cr(VI)	4.667	2.523
	Cr(III)	0.089	1.729

**Table 9:** The distribution coefficient (K_d_), selectivity coefficient (k) and relative selectivity coefficient (k’) for IIP-ANZ and NIP-ANZ.

Ion		IIP-ANZ		NIP-ANZ		*k*′
		*K*_d_ (L/g)	*k*	*K*_d_ (L/g)	*k*	
Cr(VI)/Pb(II)	Cr(VI)	0.101	–	0.066	–	–
	Pb(II)	0.014	7.181	0.019	3.556	2.019
Cr(VI)/Mn(II)	Cr(VI)	2.645	–	2.687	–	–
	Mn(II)	-0.052	-50.417	-0.219	-12.271	4.109
Cr(VI)/Ni(II)	Cr(VI)	4.781	–	1.706	–	–
	Ni(II)	10.891	0.439	13.568	0.126	3.491
Cr(VI)/Cr(III)	Cr(VI)	0.714	–	0.253	–	–
	Cr(III)	0.011	62.783	0.330	0.766	81.924

## 2 Experimental design, materials and methods

### 2.1 Reagents and materials

Potassium dichromate (K_2_Cr_2_O_7_), Sodium hydroxide (NaOH), 1,5-diphenyl carbazide, sulphuric acid (H_2_SO_4_), hydrochloric acid (HCl), acetone, nitric acid, NH_4_Cl, CrCl_3_. 6H_2_O, Ni_2_SO_4_, Mn_2_SO_4_, Pb(NO_3_)_2_, 4-vinyl pyridine (4-VP), methyl methacrylate (MMA), ethylene glycol dimethacarylate (EGDMA), benzoyl peroxide (BPO) were purchased from Merck (Singapore) and Sigma Aldrich (Singapore). Acid activated of Indonesia (Ende-Flores) natural zeolite (ANZ) was produced in our lab.

### 2.2 Preparation of Cr(VI)-poly(4-VP-co-MMA)-ANZ

To synthesize Cr(VI)-imprinted-poly(4-VP)-ANZ (IIP-ANZ), Cr(VI) was used as a template, 4-VP was used as a complex agent, MMA as a monomer agent, EGDMA as crosslinker and BPO as an initiator. Polymerization was directly done by the precipitation method, where 4-VP (8 mmol; 0.86 mL) and Cr(VI) (1 mmol; 0.3 g) were sealed in a polymerization bottle (250 mL) and added with the ethanol: acetone (2:1). This solution was kept at room temperature for 30 min to form metal-complex 4-VP-Cr(VI). After this period, EGDMA (60 mmol; 11.3 mL), 1% BOP (0.1 g in 10 mL chloroform) and ANZ (10 g) was slowly dropped into the polymerization bottle that containing the 4-VP-Cr(VI) complex. The mixture was purged with nitrogen gas for ten minutes, close a glass bottle and let polymerization in water bath thermostatic at 65 °C for one hour then the temperature was increased to 80 °C and kept constant for five hours. After polymerization, the solid polymer in sphere form was filtered and stirred in ethanol: demineralization water (70:30) for 6 h to remove the excess of the reagents. The imprint anion was removed by mixing solid polymer in 4 M HNO_3_ for 6 h. The solid polymer was filtered through 0.45 μm filter paper and a fresh nitric acid solution was added, the process was continued until satisfactory removal of dichromate was achieved and determined by UV–vis spectrophotometer. The solid polymer was then collected and washed several times with demineralization water until neutral pH was observed and drying in 55 °C. For comparison, NIP-4-VP-co-MMA-ANZ (non ion imprinted polymer) (NIP-ANZ) was also prepared using an identical procedure without the addition of dichromate ion.

### 2.3 Characterization of IIP-ANZ and NIP-ANZ

The crystalline phases of IIP-ANZ and NIP-ANZ were characterized using X-ray diffraction (PANalytical, X’pert Pro). The functional group spectra of IIP-ANZ and NIP-ANZ were characterized using Fourier transform infrared (FTIR) spectrometer (Shimadzu, FTIR 8000 Series). The surface morphology and compound of the particles were examined using scanning electron microscopy with energy dispersive X-ray (SEM-EDX) spectroscopy (JEOL, JMS 5600, Tokyo, Japan). The surface area (S_BET_), total pore volume, and pore size distribution were determined by N_2_ adsorption isotherm with the relationship between N_2_ adsorbed volume at standard conditions using Quantachrome Instruments NOVA 1200 (High-Speed Gas Sorption Analyzer Versions 10.0 – 10.03). The pH_ZPC_ and pH was determined using pH meter (spark PS-2008A) and the Cr(VI) was measured with UV–vis spectrophotometer (Shimadzu UV-1240).

### 2.4 Adsorption studies

Adsorption of Cr(VI) from aqueous solutions was investigated in batch experiments. pH effect was examined from 1–9. The pH of the Cr(VI) solution was adjusted by the addition 0.1 M HCl or 0.1 M NaOH. Ideal weight adsorbent was investigated using 0.01 g to 2.0 g. Time adsorption was studied from 0 min to 120 min. All adsorption parameters investigated were performed with Cr(VI) 14 mg/L in 50 mL of solution. For temperature effect, the investigation was conducted from 303 to 343 K with a concentration of Cr(VI) varies from 6 mg/L to 14 mg/L by addition the suspension solution in a close glass flask and stirred at constant rotation per minute for each temperature. To determine Cr(VI) in liquid phase/supernatant, solution was filtered, added a 2.0 mL diphenyl carbazide in solution, mix and Added H_2_SO_4_ solution to give a pH of 2 ± 0.5, dilute to 100 mL in a volumetric flask with water, and let stand 5 to 10 min for full colour development and measure its with UV–vis spectrophotometer at 540 nm. A number of metal ions sorbed onto the unit mass of adsorbent was calculated by the Eq. (1).

(1) $$q_{e}=\frac{\left(C_{0}-C_{e}\right)V}{m}$$

where *C*_o_ is the initial concentration of Cr(VI) in solution (mg/L), *C*_e_ is the equilibrium concentration (mg/L), *q*_e_ is the equilibrium adsorption capacity (mg/g), *m* is the mass of adsorbent (g), and *V* is the volume of solution (L).

The removal percentage of Cr(VI) can be calculated by the Eq. (2).

(2) $$\mathrm{Efficiency}\;\mathrm{of}\;\mathrm{adsorption}\left(\%\right)\;=\;\frac{C_{0}-C_{e}}{C_{0}}\times 100$$

where *C*_o_ is the initial concentration of Cr(VI) in solution (mg/L) and *C*_e_ is the equilibrium concentration (mg/L).

To study about the competitive adsorption of the target and competing ions can be used the distribution coefficient equation (Eq. (3)) [7].

(3) $$K_{d}=\frac{C_{i}-C_{f}}{C_{f}}$$

where *K*_d_, *C*_i_ and *C*_f_ represent the distribution coefficient, initial concentration and final solution concentration (mg/L). *V* and *m* are volumes of the solution (L) and mass of the IIP-ANZ or NIP-ANZ (g). Selectivity coefficient for binding of ion target in present of a competitor ion can be used Eq. (4).

(4) $$k=\frac{K_{d}\;(\mathrm{template metal})}{K_{d}\left(\mathrm{interferent metal}\right)}$$

where *k* is the selectivity coefficient of interfering metal (i.e., Pb(II) ions). A comparison of the k values of the imprinted polymer with those of metal ions allows an estimation of the effect of imprinting on selectivity. In order to evaluate an imprinting effect, a relative selectivity coefficient (*k*′) was defined as follows Eq. (5) [7], [8].

(5) $$k^{\prime }=\frac{k(\mathrm{imprinted})}{k(\mathrm{non}-\mathrm{imprinted})}$$

where *k′* is the indicator of the effect of imprinting on the selectivity of Cr(VI) adsorption on IIP-ANZ.

### 2.5 Kinetic adsorption studies

Kinetic studies were done using a given initial concentration (14 mg/L) for contact times from 0 min to 120 min. The kinetic models of pseudo first-order, pseudo second-order, Elovich, intraparticle diffusion and Bangham were used for kinetic evaluation.

### 2.6 Isotherm adsorption studies

Isotherm studies were done with Cr(VI) concentrations varies from 6 mg/L to 14 mg/L and contact time of 30 min. The isotherm models of Langmuir, Freundlich, Tempkin, and Dubinin–Kaganer–Radushkevich (DKR).

### 2.7 Thermodynamic study

The thermodynamics of Cr(VI) adsorption by IIP-ANZ or NIP-ANZ was performed at solution temperature of 303, 313, 323, 333 and 343 K and thermodynamics parameters was acquired using an estimated change in ∆*H*°, ∆*S*°, and ∆*G*° as defined in Eqs. (6), (7). The standard enthalpy change (∆*H*°) and the standard entropy (∆*S*°) for Cr(VI) sorption on IIP-ANZ or NIP-ANZ were obtained using Van’t Hoff equation.

(6) $$\ln K_{L}=\frac{{∆H}^{o}}{\mathrm{RT}}+\frac{{∆S}^{o}}{R}$$

where *K*_L_ is the adsorption coefficient from the Langmuir adsorption isotherm, ∆*H*° is the standard enthalpy change (J/mol), ∆*S*° is the standard entropy change (J/mol/K), R is the gas constant (8.314 J/mol/K) and *T* is the temperature in K. The plot of ln *K*_L_ versus 1/*T* allows determining the standard enthalpy change (∆H°) and the standard entropy (∆*S*°).

The standard Gibbs free energy change (∆*G*°) of adsorption was calculated from Eq. (6) [9].

(7) $${∆G}^{o}={∆H}^{o}-{T∆S}^{o}$$
